# Telemedicine and 5G Technologies: A Systematic Global Review of Applications over the Past Decade

**DOI:** 10.3390/bioengineering13040438

**Published:** 2026-04-08

**Authors:** Alessandra Franco, Francesca Angelone, Danilo Calderone, Alfonso Maria Ponsiglione, Maria Romano, Carlo Ricciardi, Francesco Amato

**Affiliations:** 1 Department of Electrical Engineering and Information Technology, University of Naples ‘Federico II’, 80125 Naples, Italy; alessandra.franco@unina.it (A.F.); alfonsomaria.ponsiglione@unina.it (A.M.P.); mariarom@unina.it (M.R.); framato@unina.it (F.A.); 2 Department of Engineering, University of Sannio, 82100 Benevento, Italy; fangelone@unisannio.it; 3 Research & Development Department, Outpatient Cancer Center “Emicenter”, 80020 Casavatore, Italy; danilo.calderone@emicenter.it

**Keywords:** telemedicine, 5G mobile communication, teleconsultation, robotic surgical procedures, diagnostic imaging, telemonitoring, telerehabilitation, wireless technology, health services accessibility

## Abstract

This systematic review analyzes how the introduction and progressive deployment of 5G networks have influenced the evolution of telemedicine between 2014 and 2024, focusing on their impact on performance, accessibility, and the feasibility of advanced clinical applications across the pre-COVID-19, COVID-19, and post-COVID-19 periods. The review was conducted in accordance with PRISMA guidelines and included publications retrieved from SCOPUS, PubMed, and Web of Science using a PICO-based search strategy. Studies were selected based on predefined inclusion and exclusion criteria, and extracted data included clinical parameters, network characteristics such as bandwidth and latency, geographic setting, and type of telemedicine service. A total of 45 studies met the inclusion criteria, with most published between 2020 and 2024. The most frequently reported applications were telediagnosis, particularly robotic ultrasound, followed by telesurgery and teleconsultation. The low latency enabled by 5G networks supported complex telesurgical procedures over distances exceeding 5000 km, while in ultra-remote areas, hybrid solutions combining 5G and fiber-optic networks were often adopted to ensure stable connections. The integration of robotic platforms and AI-based tools further enhanced the precision and reliability of remote procedures. Overall, 5G technology has significantly advanced telemedicine by enabling real-time, high-quality care over long distances, improving access to specialist services and supporting more equitable and efficient digital healthcare delivery, particularly in underserved regions.

## 1. Introduction

Telemedicine has recently emerged as one of the most promising solutions to greatly improve access to care and reduce health inequalities [1,2,3]. Defined as the provision of healthcare services remotely through the use of information and communication technologies, it shows great potential to address numerous challenges, such as lack of access in rural areas, treatment of chronic patients and the response to global health emergencies such as the COVID-19 pandemic, which has given impetus to the development of new remote diagnostic and treatment techniques [4,5,6,7,8].

The adoption of telemedicine has dramatically accelerated due to this global emergency, clearly showing its important role in the resilience of the healthcare system [9,10,11].

However, its effectiveness depends on the quality of the services provided and the trust patients place in them, which is why the World Health Organization (WHO) has stressed the importance of ensuring that telemedicine meets strict standards and is used responsibly. Therefore, on 15 July 2024, the WHO introduced new guidelines for improving telemedicine services, which offer guidance on various crucial aspects, such as training health professionals, protection of patient data, and integration of telemedicine into national health systems [12]. With this initiative, the WHO aimed to standardize telemedicine practices throughout Europe, promoting high standards of quality and safety [13]. Several key areas of application of telemedicine have emerged over the years, some of the most important being telehealth, which is the use of digital and communication technologies to deliver and support health services remotely, including clinical, educational, and administrative functions [14,15]; teleconsultation, a remote consultation between one or more doctors to discuss the clinical situation of a patient, based on all the clinical data/images and reports relating to the case in exam, using audiovisual communication [16,17]; telemonitoring, which allows remote detection and continuous transmission of vital and clinical parameters through the use of sensors applied to the patient [18,19]; telerehabilitation, or the remote provision of services and benefits intended to restore, improve or maintain the psychophysical functioning of people with disabilities or specific disorders [20,21]; telediagnosis, i.e., a remote medical diagnosis formulated on the basis of data transmitted by telematics [22,23]; and telesurgery, surgery practiced remotely by means of robotic technology and other telematic instrumentation [24,25,26]. However, the success of telemedicine is dependent on the quality and reliability of the telecommunication infrastructures that support these services [27]. Slow networks, unstable connections, or high latency can severely compromise the user experience and patient safety, preventing the adoption of advanced solutions. In this context, the introduction of the fifth generation of mobile networks (5G) technology represents a significant revolution. The 5G is unique in its data rates of up to 20 Gbps, twenty times higher than 4G, which stops at 1 Gbps, and a latency of less than 10 ms compared to 4G’s 50–100 ms. In terms of frequencies, 5G uses higher bands (up to 27.5 GHz), which provide higher speeds but a more limited range than 4G frequencies (800–2600 MHz) [28,29]; in addition, 5G can handle up to 1 million simultaneously connected devices per square kilometer, compared to 100,000 for 4G [30]. These features are particularly important for telemedicine, which often requires the exchange of real-time data, such as high-resolution video, diagnostic medical images, and continuous monitoring of patients’ vital parameters [31].

Between 2014 and 2024, the integration of telemedicine and 5G has seen significant growth, with applications ranging from remote diagnostics to emergency management. This period marked a transition from limited adoption in pilot scenarios to large-scale implementations, partly driven by the urgency created by the pandemic. Several recent reviews have examined the role of 5G in healthcare, mainly focusing on specific technical enablers or on emergency applications introduced during COVID-19 [32,33,34]. However, these studies generally address a limited set of use cases or concentrate on the pandemic period alone. In contrast, the present review adopts a broader and longitudinal perspective by comparing developments before, during, and after COVID-19 and by integrating literature, case studies, and cross-country implementations. The objective is to highlight the evolution of telemedicine in relation to 5G and to examine the challenges, limitations, and future opportunities of this convergence, emphasizing its contribution to a more accessible, efficient, and sustainable healthcare system.

## 2. Materials and Methods

### 2.1. Record Selection

The literature review was conducted following the methodology outlined in the Preferred Reporting Items for Systematic Reviews and Meta-Analyses (PRISMA) guidelines (Supplementary Materials: PRISMA 2020 Checklist for systematic reviews) [35]. All the methods per conducting such review were defined before gathering all the papers by using three major search engines: SCOPUS, PubMed, and Web of Science. The search query, developed based on the Patient, Intervention, Comparison and Outcome (PICO) strategy, was applied to the titles and abstracts of the selected databases. The following keywords were used: “telemedicine,” “telemonitoring,” “teleassistance,” “teleconsultation,” “televisit,” “telediagnosis,” “telesurgery,” and “telerehabilitation,” combined with at least one of the terms “5G” or “6G”.

The range of publication time spans from 2014 to 2024, with a specific focus on the pandemic and post-pandemic years. To gather all the manuscripts during the time range, sources were last consulted in March 2025. The aim is to compare the use of telemedicine during the pre-pandemic, pandemic, and post-pandemic phases. Articles were included or excluded from the analysis based on the following eligibility criteria:

Inclusion criteria:

- English research articles;
- Articles with available Full-text;
- Studies describing a telemedicine application associated with a proposed 5G telecommunication system.

Exclusion criteria:

- Reviews, Surveys and book chapters;
- Articles out of scope: studies that, although related to telemedicine or emerging technologies, did not address the role of 5G in enabling or improving telemedicine applications (e.g., papers focused primarily on robotics, or technical components without clinical or telemedical relevance)
- Studies with a strong focus on IoT network architecture, which prioritized technical aspects over telemedicine applications.

Three independent reviewers (Angelone F., Calderone D., and Franco A.) screened the titles and abstracts of all articles. Each reviewer assessed two-thirds of the studies independently. Any discrepancies in study selection were resolved through discussion and consensus.

### 2.2. Record Screening

The research articles selected based on the criteria outlined above were progressively screened. The screening process involved the extraction of relevant data for the definition of the telemedicine project presented in each record and for the definition of the telecommunication system used (5G or 6G). In particular, the data extracted were:

- Aim of the study
- Number of subjects
- Mean subject age
- Doctor–patient distance (evaluated in kilometers)
- Connection bandwidth (evaluated in megabits per second)
- Connection latency (evaluated in milliseconds)
- Duration of the performance (evaluated in minutes)
- Type of remote service

To better synthesize the studies, they were grouped into six main telemedicine applications. This was also decided according to telemedicine guidelines currently available in Italy (I servizi di telemedicina. Ministero Della Salute. 2020. https://www.salute.gov.it/new/it/tema/telemedicina/i-servizi-di-telemedicina. Accessed on 1 March 2026). Relevant data were extracted manually using a standardized data extraction form created in Microsoft Excel (Microsoft Corp., Redmond, WA, USA. Excel Version 2603). Any missing data was highlighted in the constructed tables, and the graphs were calculated solely on the available values. Each included article was reviewed twice to ensure the accuracy and completeness of extracted data. Risk of bias in the studies was assessed by a single reviewer (Ricciardi C.).

## 3. Results

### 3.1. Study Selection

**Figure 1:**
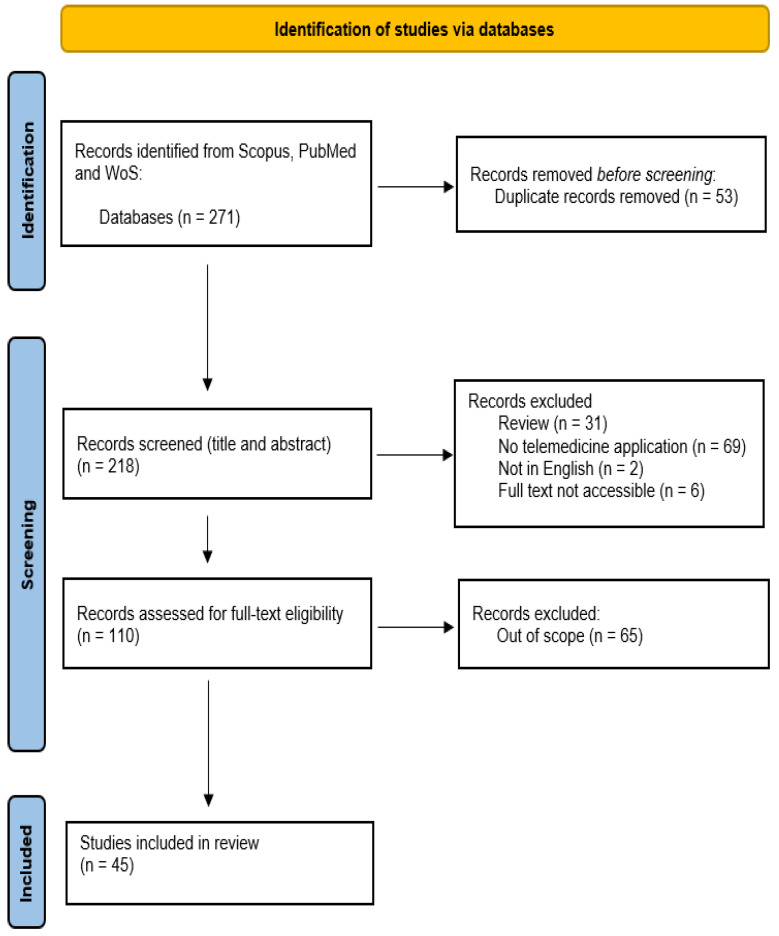
PRISMA 2020 flow diagram of the study selection process.

A PRISMA flow chart diagram was prepared to illustrate the selection process of included papers, as shown in Figure 1. The search yielded a total of 271 records; of these, 45 met the selection criteria and were included in the systematic review. Subsequently, the data present in the extraction Table 1 were summarized to perform a qualitative and quantitative synthesis and to organize the information into sections.

### 3.2. Characteristics of the Included Studies

#### 3.2.1. Temporal Distribution

**Figure 2:**
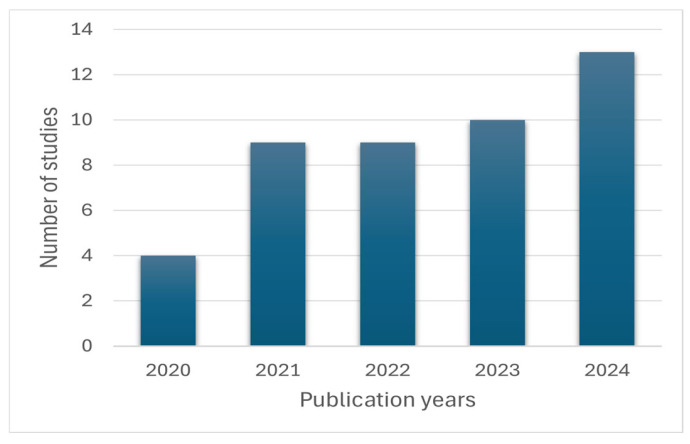
Temporal distribution of included papers.

Although the search for papers covered the last 11 years, the screening highlighted a distribution of articles relating to telemedicine applications only in the last five years, from 2020 to 2024, with a peak in 2024 with 29% of articles included, 22% in 2023, 20% in 2022 and 2021, and 9% in 2020, as shown in Figure 2.

#### 3.2.2. Main Telemedicine Applications

**Figure 3:**
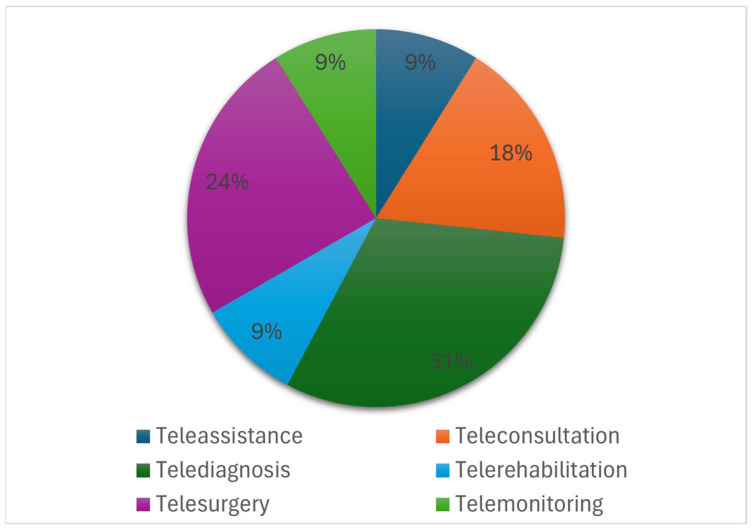
Main telemedicine applications in the included papers.

The screening of the papers showed that most of the papers included in this review dealt with telediagnosis applications (31%), followed by telesurgery (24%), teleconsultation (18%), telerehabilitation (9%), telemonitoring (9%) and teleassistance (9%), as shown in Figure 3. It is also interesting to note that almost all the articles on telediagnosis applications involved ultrasonography, except for only one that dealt with endoscopy.

#### 3.2.3. Geographical Distribution

**Figure 4:**
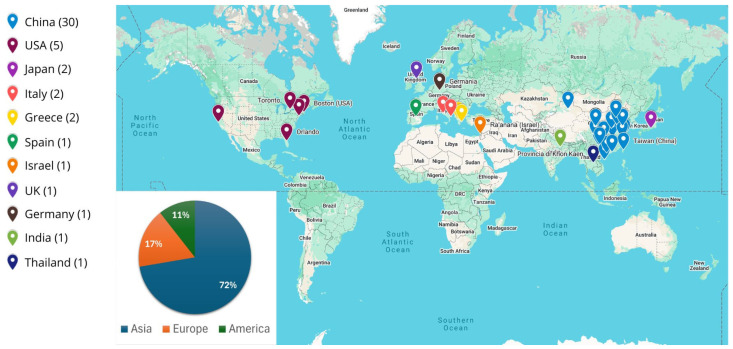
Geographical distribution of telemedicine applications in the included papers.

As can be seen in Figure 4, most of the telemedicine applications analyzed in this review work were carried out in Asia, mainly in China (85% of Asian studies), Japan (6% of Asian studies), and Israel, Thailand and India (each 3% of Asian studies), followed by Europe (Greece, Italy, Germany, UK and Spain) and America (Canada and USA).

#### 3.2.4. Robots

Robotic systems are frequently used in telemedicine, particularly in the fields of telesurgery and telediagnosis [26,79]. In telesurgery, four articles reported the use of the Micro Hand S robotic system (Shandong Weigao Surgical Robot Co., Ltd., Weihai, China), while other robots included the CorPath GRX (Corindus, a Siemens Healthineers Company, Waltham, MA, USA), the KD-SR-01 (KangDuo Surgical Robot Co., Ltd., Shanghai, China), the TiRobot system (Tinavi Medical Technologies Co., Ltd., Beijing, China), the Toumai (MicroPort MedBot Group Co., Ltd., Shanghai, China), the Edge Medical Robot MP1000 (Edge Medical Robotics Co., Ltd., Beijing, China), the iRes2 excimer laser platform (iVIS Technologies, Taranto, Italy), and the MicroPort MedBot robotic platform (MicroPort MedBot Group Co., Ltd., Shanghai, China). For telediagnosis, the most frequently used robot was the MGIUS-R3 (MGI Tech Co., Ltd., Shenzhen, China), mentioned in 8 out of 14 articles. Other systems included the M5-700 robotic arm (Megarobo Technologies Co., Ltd., Beijing, China), the DOFs robotic arm, and 5G-based robotic ultrasound platforms.

#### 3.2.5. 5G Network Provider

Of the 45 articles included in this review, most did not specify the 5G network provider used. Of the remaining few articles, the most frequently reported vendor was China Telecom (n = 4), followed by NTT Docomo (n = 2), Vodafone, including Vodafone Greece (n = 2), Verizon Wireless (n = 1), China Unicom (n = 1), and China Mobile Communications Group Co., Ltd. (n = 2), which includes regional subsidiaries such as China Mobile Shandong and Guangdong.

## 4. Discussion

The literature reviewed shows a sharp increase in publications after 2020, as illustrated in Figure 2, highlighting the rapid acceleration in the adoption of telemedicine during and after the COVID-19 pandemic. This trend suggests that the global health emergency has accelerated the development and implementation of digital health solutions based on advanced communication technologies. The distribution of telemedicine applications observed in the included studies (Figure 3) highlights how 5G technologies were initially explored primarily in clinical contexts requiring high network performance, such as telediagnosis and telesurgery, where high data transmission speeds, low latency, and reliable connections are essential. At the same time, the presence of studies on teleconsultation, teleassistance, telerehabilitation, and telemonitoring highlights 5G’s potential to support more common healthcare services as well, improving access to care and patient monitoring. These developments could also contribute to more efficient healthcare delivery by enabling faster data exchange, facilitating real-time collaboration between specialists, and supporting more timely clinical decision-making. From a geographical perspective (Figure 4), Asian countries, particularly China, appear to be making a significant contribution and seem to play a leading role in the testing and implementation of 5G-based healthcare solutions. Although the search strategy also included the term “6G” to capture potential emerging developments, no relevant studies on 6G-based telemedicine were identified during the period under consideration. This reflects the current stage of technological development, in which 6G remains largely at the conceptual level. In light of these considerations, the discussion of the included studies is organized in the following sections according to the main application areas of telemedicine.

### 4.1. Telediagnosis

The 5G applications of telediagnosis, most explored in the screened papers, involve ultrasonography. Ultrasound (US) transmits high-frequency sound waves (1 to 5 MHz) through a probe to produce images of the inside of the body. To propagate, ultrasound needs a substrate and depending on the substrate will propagate with a different wave propagation speed. Acoustic Impedance is defined as the intrinsic resistance of substrates to being passed through by ultrasound. It conditions the propagation speed in the tissues and is the principle behind the ultrasound technique. Ultrasound is widely used because it is a non-invasive diagnostic technique, fundamental in all screening programs, for its real-time performance, and low price. The need to develop and analyze teleultrasonography applications arises mainly from: i) reaching rural and remote areas, where often there is a lack of specialist doctors, and ii) protecting operators and physicians in infectious disease situations, such as COVID-19. 5G communication systems, thanks to their high-capacity, high-speed communication with low latency, transform and provide a further boost to remote diagnosis. In teleultrasound, transmission speed and high image quality are important. Saeki et al. [61], based on a qualitative evaluation made by 3 sonographers regarding the video delay time, concluded that for 5G communication, the delay for 5G communication was “acceptable” or “fairly acceptable,” which was better than that for LTE communication, where all physicians agreed that the delay was “not acceptable”. This shows how 5G is fundamental to the growth and diffusion of telemedicine on a large scale [32,33,34]. This qualitative advantage is reflected quantitatively in several other works. For instance, He et al. [58] observed high diagnostic reliability in breast imaging with excellent interobserver agreement (ICC = 0.795–1.000), comparing the evaluation of the characteristics and the BI-RADS classification of the nodules reported in the conventional US and the remote US. They assessed the security, longevity, imaging quality of ultrasound, reliability, and acceptability of telerobotic ultrasound using 5G technology in identifying breast conditions to support an efficient screening initiative, even in rural and isolated locations. Instead, Ren et al. [60] evaluated the feasibility and satisfaction of patients in an ultrasound examination in underserved rural areas. They reported that on 546 subjects, of which 115 responded to the satisfaction questionnaire, finding that 98% of individuals were at ease during the abdominal and urogenital evaluation, while 114 participants were comfortable interacting with the sonographer via a remote audio-video system. Additionally, merely 2% expressed any fear regarding the use of the robotic system. The duration of the consultation, the quality of the images, and the safety were instead evaluated by Duan et al. [62], again in the abdominal US, on 32 subjects included in the study. The mean score assigned to image quality was 4.73, which, on the internationally accepted five-point absolute scale, reflects an image of good quality. Concerning safety, vital signs did not change after the teleultrasound examination, and no complications attributable to the examination were reported. Liang et al. [56] consider an important aspect as the examination duration. They highlight how, although the complete examination rate was not significantly lower than the traditional approach, telerobotic abdominal ultrasound had longer examination times than traditional ultrasound (9.87 ± 4.41 vs. 2.43 ± 0.42 min), when there is a distance of 106 km between the patient site and the doctors’ site. This aspect was also confirmed by a study conducted by Zhang et al. [57] on 401 patients on a shorter distance (72 km), in which it is highlighted that the telerobotic US examination duration took longer than the conventional US (12.54 ± 3.20 min vs. 7.23 ± 2.10 min).

In COVID-19 cases, robot-assisted teleultrasound and remote consultation have proven to be fundamental in solving the problem of early cardiopulmonary assessment, while still guaranteeing the safety of operators. Cardiopulmonary evaluation generally includes lung ultrasound, echocardiography, and evaluation of blood volume. Several studies [63,64,66,67] have presented case studies in which the feasibility of using teleultrasound in cases of COVID-19 is confirmed to monitor cardiopulmonary function and intervene quickly. In this study, it was shown that lung US provides clinical diagnostic information comparable to chest CT, which is considered best practice. However, chest CT has limitations as it is difficult to perform in critically ill patients who cannot move, children and pregnant women cannot be exposed to radiation, the closed environment of CT may contribute to the spread of COVID-19, and it cannot reach remote areas.

Interestingly, all robotic ultrasound studies use the MGIUS-R3 (MGI Tech Co., Ltd., Shenzhen, China) robotic ultrasound system, which integrates remote robotic control, ultrasound examination, and audiovisual communication. MGIUS-R3 (MGI Tech Co., Ltd., Shenzhen, China) is made up of two main parts: the doctor-side subsystem and the patient-side subsystem. The doctor-side subsystem features an ultrasound display system, an audio-visual communication system, and a control system. The patient-side subsystem includes an ultrasound imaging system, an audio-visual communication system, and a robotic arm with six degrees of freedom positioned next to the patient. These components are connected through a 5G network, which offers a download speed of 930 Mbps and an upload speed of 132 Mbps.

Duan et al. [62] present an innovative remote robotic ultrasound system featuring a robotic arm on the patient’s side, specifically the UR5 collaborative robot (Universal Robots A/S, Odense, Denmark). This robot possesses six degrees of freedom and is equipped with a force sensor to carry out movement commands from the physician’s side. It can operate using wired connections as well as 4G and 5G wireless networks. The system underwent trials with 95 participants focusing on the thyroid, carotid arteries, and abdominal area, showing that both healthcare providers and patients view it as dependable and secure. Furthermore, the Structure Similarity Index metric (SSIM) is calculated between the image seen in the patient subsystem and the one seen in the doctor subsystem, showing values greater than 0.99 in all cases.

The growing use of 5G robotic ultrasound in healthcare requires standardizing methods for assessing participant satisfaction. In the reviewed studies, satisfaction is mainly based on the evaluation of video transmission delay or image quality. Beyond image performance, satisfaction studies also provide comparative insight. Han et al. [55] showed that 84% of participants were satisfied with 5G-assisted remote ultrasound examination and its influencing factors using structural equation modeling (SEM). From the 201 completed questionnaires, which addressed five aspects (efficiency, perception of the exam, communication, value, and availability), participants reported a mean satisfaction score of 45.43 ± 11.60. Path analysis indicated that satisfaction was positively influenced by exam effectiveness, exam perception, communication perception, and perceived value. Furthermore, satisfaction itself showed a direct positive association with willingness to take the exam, with a standardized path coefficient of 0.260. It also emerged that significant differences were present between different levels of education and between patients with different body mass indexes (*p*-value < 0.05) in the dimensions of perception of the examination, perception of communication, perception of value, and propensity to undergo the examination.

The only other 5G-based telediagnosis applications, other than teleultrasound, have been explored by Zhang et al. [59] and Nia et al. [54], who evaluated the feasibility and safety of a novel 5G-based remote capsule endoscopy system for real-time remote gastrointestinal examinations. In [59], the complete visualization rate of the stomach and small intestine, safety assessment, and network latency time of remote examinations were analyzed. 20 participants underwent conventional and remote practice. The stomach and small intestine could be completely visualized in both groups (*p* > 0.999). The network latency of the remote group was 19.948 ms (median value). For endoscopy, as with ultrasound, the gastric examination time was slightly longer (8.96 vs. 8.92 min, *p* = 0.234). All participants found remote endoscopy acceptable and necessary. In [54], a feasibility study was conducted on patients affected by colorectal cancer based on the carbon footprint analysis and on structured questionnaires and interviews administered to 25 participants. 88% of participants expressed satisfaction with the remote practice. It also emerged that the carbon footprint associated with the administration of the practice at home was lower than the practice administered in a clinical context but could improve in an optimized context.

### 4.2. Telesurgery

The 5G network, enabling real-time data transmission and rapid communication among doctors and patients, promotes also the remote performance of complex surgeries. In this review, 42.86% of the studies involved surgical simulation, while 57.14% reported actual surgical procedures performed on patients.

In surgical simulation applications, the primary objective was to test the efficacy, safety, and stability of ultra-remote telesurgery under 5G conditions, and the studies consistently demonstrated that, despite differences in surgical models, operative tasks, and communication distances, 5G connectivity was generally capable of supporting uninterrupted robotic manipulation. In the study by Zheng in 2020, a swine model was utilized to conduct four laparoscopic operations, which included left nephrectomy, partial hepatectomy, cholecystectomy, and cystectomy, all carried out with the MicroHand surgical robot (Shandong Weigao Surgical Robot Co., Ltd., Weihai, China) [52]. The separation between the operator and the patient locations was about 3000 km. The average network delay, surgical duration, amount of blood loss, and complications occurring during surgery were documented for assessment. The average network delay was recorded at 264 ms, the total duration of the operation was 2 h, the total volume of blood loss was 25 mL, and no complications arose during the procedures. This illustrates the capability of conducting laparoscopic surgery in ultra-remote locations, facilitated by the 5G network.

Compared with this, Madder et al. [51] provided a more granular comparison by testing the performance of robotic telestenting for transcontinental (4964.826 km) and regional distances (331.525 km), and also between wired and 5G-wireless networks. Specifically, an attempt was made to perform percutaneous coronary intervention (PCI) utilizing a commercial endovascular simulation system (ANGIO Mentor, Simbionix, Littleton, CO, USA) along with the robotic system Cor-Path GRX (Corindus, a Siemens Healthineers Company, Waltham, MA, USA). The analysis revealed that the transcontinental design experienced greater latency compared to the regional design in both wired networks (121.5 ± 2.4 ms vs. 67.8 ± 0.9 ms; *p*-value < 0.001) and on 5G wireless networks (162.5 ± 1.1 ms vs. 86.6 ± 0.6 ms; *p*-value < 0.001), and the increased distance did not correlate with notably different procedure durations when compared to the regional model, for both cases conducted on wired networks (4.1 ± 1.9 min vs. 9.0 ± 7.1 min; *p*-value = 0.05) and for 5G wireless networks (3.0 ± 0.6 vs. 6.3 ± 1.2; *p*-value = 0.36). Nevertheless, it became clear that wired network connections exhibited reduced latency compared to 5G wireless connections, even in the regional model (67.8 ± 0.9 ms vs. 86.6 ± 0.6 ms; *p*-value < 0.001) and the transcontinental model (121.5 ± 2.4 ms vs. 162.5 ± 1.1 ms; *p*-value < 0.001).

In a contrasting scenario focused on task-level performance, Moustris et al. [49] examined the effects of the 5G network on surgical performance (cutting, dissection, pick-and-place, and ring tower transfer) during a telesurgery simulation in a training phantom where the surgeon and the robot are separated by almost 300 km. The surgical robot used is DDelta (National Technical University of Athens, Athens, Greece; Lublino Accrea Engineering, Pisa, Italy). A latency of 18 ms was recorded for motion commands and 350 ms for video delay, allowing the surgeon to rate the usability of the system as neutral or positive. An even more extreme test of distance was reported by Patel et al. [26], who tested the feasibility of a telesurgical approach for distances greater than 10,000 km (Orlando-Shanghai) by performing ten radical nephrectomies and two partial nephrectomies on five animals with the MicroPort MedBot robotic platform (Shanghai MicroPort MedBot Group Co., Ltd., Shanghai, China). It turns out that over such long distances, hospital fiber optic networks provided greater signal stability and improved latency compared to 5G networks: a median of 250 ms with hospital fiber optics versus 375 ms with public 5G. The fact that all procedures were still completed without compromising animal survival highlights that latency tolerances vary depending on the type of robotic platform and surgical workflow.

Recognizing latency as the central determinant of telesurgical safety, Li et al. [47] compared two different network latency management systems in 5G telesurgery, conducting 20 telesurgery simulation trials and 15 remote adrenalectomy procedures. At present, the gold standard technique for assessing latency in telesurgery is the “Ping (Packet Internet Groper) command”, which includes the network delay measurement component known as “Round Trip Time” (RTT). This refers to the time it takes for a data packet to move from the sender’s location to the recipient’s location and back. Although the Ping command lacks an appealing interface, it is capable of measuring several IP addresses simultaneously with a single command. However, this process entails complex operations that may pose challenges for individuals who are not professionals in the field. Since surgeons or nurses will be responsible for monitoring network latency, it is important that the monitoring system is easy to use, and it is also important to add a warning function in case of high latency. Consequently, the authors in [47] created the Telemedicine Network Latency Management System for healthcare professionals engaged in telesurgery, addressing the specified requirements. In the examination of 15 patients with adrenal tumors, treated using the “MicroHand S” surgical robot, where the average separation between the operating surgeon and the primary hospitals was 250 km, the Telemedicine Network Latency Management System proved to be stable throughout the entire monitoring period. Furthermore, there was no notable variation found between the RTTs captured by the Telemedicine Network Latency Management System and the ones obtained through the traditional “ping command” method in all experiments performed. Instead, to test the alarm, a 4G network was used in simulated cases, as it was difficult to find a high network latency with the 5G network. It was found that when the instantaneous RTT exceeded 100 ms, the color of the real-time delay changed to red, and a rapid alarm sounded at the same time, warning the surgeon to stop manipulation.

Regarding 5G telesurgery applications in patients, a series of case studies have been proposed in the literature. Tian et al. [53] reported 12 spinal surgeries with the TiRobot system (Tinavi Medical Technologies Co., Ltd., Beijing, China) at an average latency of 28 ms, enabling a single surgeon in Beijing, China, to operate across several remote hospitals, significantly lower latency than in the long-distance laparoscopic studies, suggesting that regional networks provide much more stable conditions for delicate orthopedic procedures. Similarly, Yang et al. [50] used MicroHand S (Shandong Weigao Surgical Robot Co., Ltd., Weihai, China) for a radical cystectomy at nearly 3000 km, reporting 254 ms latency (compared to 247 ms on wired connection), while Li et al. [47] used the same robot for nephrectomy across shorter distances (187 km) and achieved lower delays (200 ms) and a 100% success rate. These differences reinforce that even within the same robotic platform, distance and infrastructure strongly affect latency but may not necessarily compromise surgical outcomes. In another clinical case, the MP1000 robot (Edge Medical Robotics Co., Ltd., Beijing, China) [45] enabled a prostatectomy across 1300 km with only 22 ms latency, significantly outperforming the latency ranges observed in long-distance MicroHand S surgeries, suggesting that robot design and network configuration can dramatically influence performance.

More recently, the unique case of 5G telesurgery was demonstrated by Alessio et al. [46], who performed a combined topography-guided transepithelial photorefractive phototherapeutic keratectomy (PRK-PTK) using the iRes^®^2 excimer laser platform (iVIS Technologies, Taranto, Italy) between two different on-site rooms, demonstrating its effectiveness again with a latency that, due to the fast 5G Internet connectivity, is reduced from 0.27 to 0.01 s [46]. Building on the progression from simulation to clinical feasibility, the first robot-assisted lobectomy exceeding 5000 km was reported by Tian et al. [44] using Toumai^®^ endoscopic surgical robotic system (MicroPort, Shanghai, China) with 5G technology, with a latency of 100 ms and no connection drops. Overall, across all telesurgery studies, comparisons demonstrate that while latency increases with distance and depends on both network and robot type, 5G consistently provides sufficient stability for many surgical tasks, and successful outcomes appear more influenced by procedural complexity, robot design, and latency-management tools than by distance alone.

### 4.3. Teleconsultation

Mixed reality, supported by dependable low-latency connections from the 5G network, has significantly contributed to synchronous teleconsultation. It is capable of merging the 3D hologram with the real world that users perceive, creating a two-way interactive connection between the virtual and physical environments to improve the feeling of reality and space. Lu et al. [40] tested the Visual 3D mixed reality system (VisualMedTech Co., Ltd., Beijing, China) in different clinical scenarios of orthopedic surgery, including preoperative communication and remote consultation. The adoption of 5G networks enabled the transmission of holographic projections and real-time interaction between surgeons and remote specialists via an audiovisual platform, ensuring that both parties had access to identical views and surgical guidance. After the procedures, surgeons completed a Likert-scale survey to evaluate aspects such as the quality of communication, mutual understanding, spatial perception, and the usefulness of mixed reality tools. The results indicated lower scores in the areas of mental load, time management, performance, and frustration compared to conventional approaches, while still highlighting clear advantages in terms of understanding and communication. Compared to this moderate cognitive burden, the study by Zhang et al. [41] demonstrated that head-mounted mixed reality devices (HoloLens, Microsoft Corp., Redmond, USA) could support neuroendoscopic telecollaboration across 2489 km with only 23 ms latency for audio/video transmission, indicating that when latency is extremely low, real-time depth perception and spatial alignment become more seamless and less cognitively taxing.

Even more extreme distances were explored by Din et al. [42], where a GOOVIS virtual reality headset (GOOVIS Technology Co., Ltd., Shenzhen, China) supported keratoprosthesis implantation over roughly 9354 km (distance Toronto-Israel). Despite the far greater communication distance compared to [40,41], surgeons reported excellent audio/video quality without perceived delay, suggesting that VR-based teleconsultation may be more resilient to distance-related latency than mixed reality systems due to simpler visual overlays and lower spatial synchronization requirements.

Beyond visualization, artificial intelligence (AI) can further compensate for bandwidth and noise limitations, as shown by Xie et al. [43]. AI can help in image post-processing to address the high memory and transmission consumption, image noise, and inefficient surgical planning discussions [80,81]. The authors propose a framework for surgical telementoring in congenital heart disease that includes three modules: an AI-based image compression module, an AI-based image denoising module, and an AI-based CT image segmentation module. Subsequently, virtual operation training was implemented using virtual reality technology. The operation was successful, and the transmission rate for video streams had a latency of 30 ms, over a distance of approximately 338 km. Interestingly, despite the extensive and well-established literature on AI-based medical image denoising and processing [82,83,84,85,86], only a limited number of studies on telemedicine and 5G-enabled healthcare explicitly leverage these techniques for image processing in remote clinical workflows. This research gap is particularly noteworthy, especially considering the crucial role of image quality in bandwidth-constrained and latency-sensitive telemedicine applications.

Finally, remote teleconsultation can serve as a training purpose, which is especially necessary for ultrasound imaging, as many rural and peripheral hospitals or medical centers lack specialized personnel [37]. The researchers illustrate the practical application of a robotic teleultrasound diagnostic system powered by 5G for percutaneous nephrolithotomy, aiding less experienced surgeons during procedures on 15 individuals. Despite the very long distance between remote experts and local operators (>5800 km), the system achieved an overall delay of 177 ms, which, while higher than the latency reported in [43,59], was still sufficient for novice guidance, highlighting that acceptable latency thresholds depend strongly on the complexity and temporal sensitivity of the task. Together, these studies indicate that 5G-enabled teleconsultation is adaptive across mixed reality, virtual reality, AI-assisted platforms, and long-distance training scenarios, with differences in latency tolerance and cognitive load emerging as key factors influencing usability across applications.

### 4.4. Telerehabilitation

Telerehabilitation provides comprehensive rehabilitation services remotely using telecommunications technology as a delivery medium. It requires real-time data transfer, such as high-quality images and videos, vital signs monitoring and transmission, and audio communications. These demands highlight the importance of 5G’s high bandwidth and low latency, particularly for motion-capture systems and wearable sensors that must transmit precise biomechanical and physiological information without delay. The studies in [61,77] demonstrated that 5G substantially improves the subjective quality and acceptability of remote rehabilitation sessions: six of seven participants rated 4K video quality at 15 Mbps over 5G as “good,” outperforming LTE connections, and communication quality was reported as comparable to in-person sessions. When contrasted with the more clinically intensive context of exercise-based cardiac rehabilitation (CR), these findings suggest that 5G’s benefits scale with both task complexity and bandwidth requirements. While the mixed ultrasound–rehabilitation scenarios in [61,77] emphasized visual fidelity and interaction quality, the home-based CR model in [76] extended these advantages to physiological monitoring and therapeutic adherence. In that study, patients equipped with a 5G-enabled IoT rehabilitation system demonstrated significantly higher exercise capacity (MET and VO2 max, *p* < 0.001) and more favorable atherosclerotic profiles (lower LDL, total cholesterol, and triglycerides, and higher HDL, all *p* < 0.05) compared to the hospital-based CR group. This contrast highlights that while 5G improves user experience in standard telerehabilitation tasks, its real transformative impact emerges in longitudinal programs like CR, where reliable high-frequency data transmission supports continuous supervision and greatly enhances adherence, an effect not observed to the same extent in LTE-based or partially connected systems. Overall, comparisons across the studies show that 5G consistently enhances video quality, communication stability, and physiological monitoring across heterogeneous rehabilitation contexts, with the strongest clinical benefits occurring in programs that rely on sustained engagement and dense data flows.

### 4.5. Teleprogramming

For subjects with implantable devices, postoperative consultation is not sufficient, and they require regular scheduled postoperative follow-up visits to obtain the most suitable parameters and configuration. However, regularly going to the hospital for check-ups takes a lot of time and costs a lot of money. Thanks to the advancement of 4G and 5G networks, it has become feasible to use wireless devices that enable remote settings, simplifying the process of post-surgery programming for individuals with implanted devices. Several studies are present in the literature, especially relating to the COVID-19 and post-COVID era. Han et al. [78] presented a case of remote programming for spinal cord stimulation (SCS) for chronic pain patients. The programming is done through a remote system developed by Beijing PINS Medical Co., Ltd. (Beijing, China) allows doctors to implement SCS system programming in patients anytime and anywhere with the available network through a PINS-App (Beijing PINS Medical Co., Ltd., Beijing, China). The implanted pulse generator has the ability to connect wirelessly to the participants’ mobile phones using Bluetooth, which simultaneously links to the doctors’ computers for remote management. Medical professionals were able to modify stimulation settings, check the battery condition, evaluate electrode resistance, and perform device troubleshooting via the teleprogramming module accessed through the doctor’s client. It was revealed that 68.8% of the individuals involved requested remote monitoring, and 96.7% of them were pleased with the service. Even for patients with cardiovascular implantable electronic devices (CIED), remote programming via a real-time 5G cloud follow-up platform has gained favor with patients, with 89.8% choosing cloud follow-up for further device management [39]. Another interesting aspect is the impact of remote programming on the time spent by medical staff. While Tong et al. reported an average remote session duration of only 5.4 ± 3.5 min, Seiler et al. found that traditional face-to-face device checks require 37.8–51.0 min for therapeutic devices and 39.9–45.8 min for diagnostic instruments such as insertable cardiac monitors (ICM) [87]. Even conventional remote transmissions (9.4–13.5 min for therapeutic devices; 11.3–12.9 min for diagnostic ones) remain longer than the 5G-supported cloud sessions described in [39], suggesting that the transition from earlier remote systems to real-time 5G platforms yields an additional reduction in clinician workload. Overall, comparisons across these works demonstrate that 5G-enabled teleprogramming not only matches traditional follow-up in clinical functionality but also significantly improves patient satisfaction and reduces utilization of healthcare resources.

### 4.6. Telemonitoring

Telemonitoring primarily addresses the challenge of the rapidly growing elderly population, with the resulting increase in chronic diseases, by improving the monitoring of parameters for remote patients. Although e-health systems are increasingly leveraging advanced technologies, developing a computational framework that supports collaborative healthcare remains a challenge. Humayun et al. [70] proposed a 5G-based multi-agent framework for patient monitoring, evaluated using data from four patients with chronic diseases. The framework consists of four agents: MADO (Mobile Agent Doctor), which detects vital signs via wearable sensors; DAH (Doctor Agent in Hospital), which forwards only abnormal cases to specialists; and NA (Nurse Agent) and PA (Pharmacy Agent), which are activated by specialists’ prescriptions to provide care and medications. The multi-agent architecture proposed by Humayun et al. [70] demonstrated how 5G can streamline communication flows by forwarding only abnormal cases to specialists, thereby reducing clinician burden and ensuring timely intervention. However, scientific research has shown that people spend up to 87% of their time indoors, and this percentage is even higher in patients with chronic respiratory diseases who tend to lead a sedentary lifestyle. Therefore, in the study by Angelucci et al. [71], the sensor system also features an environmental sensor (uHoo, Kwun Tong, Hong Kong) as well as the Airgo™ band (MYAIR Inc., Boston, MA, USA; MyAirGo Italy Srl, Milan, Italy) and the SAT-300 fingertip pulse oximeter (Contec Medical Systems Co., Ltd., Qinhuangdao, China). Furthermore, thanks to 5G technology, they were able to introduce continuous monitoring rather than random monitoring. The combination was able to compare breathing patterns from one activity to another. Significant differences were specifically identified in tidal volume and minute ventilation when comparing horizontal and vertical postures (*p*-value < 0.001) and between vertical postures and dynamic activities (*p*-value < 0.001); respiratory rate shows statistically significant differences between horizontal and vertical postures (*p*-value < 0.001). More recently, Srichan et al. [68] explored a telemedicine health kiosk system connected using a 5G network and integrated with noninvasive biosensors, comparing performance to a control group and showing notable enhancements in average fasting blood glucose (from 148 to 130 mg/dL) and systolic blood pressure (from 152 to 138 mmHg), among individuals with non-communicable diseases. These results highlight how 5G-enhanced monitoring can translate into measurable outcomes in chronic disease management, beyond the primarily descriptive physiological monitoring reported in [71]. The idea is to develop a “hospital without walls” as already tested by the Second Guangdong Provincial General Hospital [69], which has developed a 5G-based Smart Home Ward that brings healthcare services into people’s homes. This approach has demonstrated its effectiveness in managing four specific illnesses, such as heart disease, stroke, Parkinson’s disease, and Alzheimer’s disease. However, although telemonitoring systems increasingly support continuous and multi-parametric data acquisition, the integration of advanced artificial intelligence techniques into remote clinical workflows remains limited, revealing a gap between methodological advances in medical data analysis and their practical adoption in telemedicine [88,89]. Overall, across these studies, 5G-enabled telemonitoring shows a consistent pattern: while early frameworks focused on communication efficiency or specific physiological metrics, more recent systems leverage 5G to achieve continuous, multi-parameter, and clinically actionable monitoring, collectively demonstrating that 5G improves both the depth and the clinical impact of remote patient assessment.

### 4.7. Teleassistance

Teleassistance is the support a telemedicine network provides from one clinical professional to another facing an emergency health situation. Mixed reality enables scenarios in which the emergency personnel wear glasses capable of transmitting audio, video and data streams to medical specialists stationed in a hospital. This requires a large data stream to be transmitted bidirectionally over a reliable network over any distance. The 5G network has enabled this, thanks to its ultra-high-speed and low-delay transmission, creating new opportunities to manage emergencies. Specifically, Garcìa et al. [74] examine a case study involving a homeless patient experiencing syncope caused by a third-degree atrioventricular block. They utilize Health-5G, a comprehensive system put forward by the authors that features a flexible structure built upon a decentralized deployment computing model, consisting of 4 interrelated applications: Health-5G Holo, utilized by the emergency team for communication and receiving guidance through the Microsoft Hololens 2™ MRI device (Microsoft Corp., Redmond, WA, USA), Health-5G Desktop, installed on the computer that operates remote medical tools, Health-5G Client, functioning on a device integrated into the emergency team and tasked with gathering information from the physician, Health-5G Server, hosted in the cloud and tasked with transmitting and receiving data for other applications. This application showed a latency of 300 ms. Furthermore, a videoconferencing performance test was carried out, which is especially essential in the case of the HoloLens 2 headset, as a low number of frames per second (FPS) could induce dizziness, headache, nausea, and other problems, which absolutely cannot occur in the context of a medical emergency. These tests showed that performance is adequate at 60 FPS or higher. In comparison, Xiang et al. [75] demonstrated that even without mixed reality, a 5G intelligent first-aid platform can dramatically accelerate patient management: remote specialists monitored and guided the care of a 68-year-old patient with chest pain during ambulance transport, enabling direct transfer to the catheterization laboratory and treatment within two minutes of hospital arrival. This contrasts with [74], where mixed-reality support focused on situational awareness and remote visualization, while [75] emphasized workflow optimization and bypassing intermediate steps in the emergency chain. The benefits of 5G-based communications can be further enhanced if AI is also used. In particular, the feasibility and reliability of 5G internet networks based on AI and mobile ultrasound in an integrated pre-hospital ambulance for telediagnosis, severity classification, triage calculation, prognosis and treatment planning to support Traumatology and Emergency Surgery cases were evaluated through the development of Cobot PROMETHEUS III (Program of Excellence 2014–16 research project, Greece) [72]. These aspects are significant in view of the effective management of the entire clinical workflow. Complementing ground-based scenarios, Han et al. [73] demonstrated that 5G networks also enhance airborne rescue operations by enabling real-time transmission of high-definition images and continuous monitoring of vital signs during flight. Their findings suggest that airborne telehealth benefits even more from 5G than ground-based systems, as it enables extended intervention ranges up to 600 km and significantly reduces response times. Overall, comparisons between these studies indicate that, although mixed reality, mobile ultrasound, AI-enabled triage, and airborne telemedicine present different requirements in terms of network speed and stability, 5G consistently provides the low latency and high bandwidth necessary for timely, coordinated, and clinically meaningful telehealth.

## 5. Conclusions

This systematic review examined the evolution of telemedicine between 2014 and 2024 in relation to the progressive integration of 5G networks, comparing applications before, during, and after the COVID-19 pandemic. The longitudinal analysis confirms that 5G expansion has not simply improved existing services but has also enabled the transition from small pilot projects to mature, scalable clinical implementations across multiple settings. Before 5G, the evolution of advanced telemedicine was limited by several barriers, including high latency, bandwidth limitations, unstable wireless connections, and the poor quality of audiovisual streams. These factors have hindered real-time applications such as telesurgery, robotic telediagnosis, mixed reality teleconsultation, and continuous high-resolution monitoring. During the pandemic, the urgent need for remote care accelerated experimentation, but many systems still relied on LTE or hybrid infrastructures that could not guarantee the reliability, responsiveness, and data transmission speed required for highly urgent or image-intensive procedures. The studies included in this review demonstrate that the introduction of 5G has helped overcome many of these barriers, offering low latency, high bandwidth, and more stable long-distance connections, enabling clinical scenarios previously unattainable at scale. Robotic teleecography has demonstrated high diagnostic accuracy even in remote settings; telesurgery has become technically feasible over distances exceeding 5000 km, although wired fiber optic networks remain more efficient for very long-range procedures; and mixed reality, AI-enhanced imaging, and IoT-based monitoring systems have benefited from faster data transmission. At the same time, telemonitoring, telerehabilitation, and telehealth have demonstrated how 5G can support seamless, real-time, multisensory data integration in both in- and out-of-hospital care. Despite the progress noted, it is important to acknowledge certain limitations. First, the reporting of key technical variables in the included studies (such as latency, bandwidth, and doctor–patient distance) was often incomplete or inconsistent. Since this information was sometimes not reported in the publications, it was not always possible to make direct comparisons between studies, with potential implications for the robustness of the overall synthesis. Furthermore, to provide a comprehensive overview of 5G-enabled telemedicine, studies involving heterogeneous technologies, network configurations, and clinical applications were analyzed together. Although this approach allows for a broader view of the current state of the field, it also introduces a degree of variability that may limit the comparability and interpretation of the results. Moreover, when interpreting the results, some characteristics of the available literature must be considered. The geographical distribution of the included studies is uneven, with a strong concentration in Asian countries, particularly China, which may limit the generalizability of the findings to other healthcare settings. In addition, many studies represent pilot implementations or technology demonstration projects, rather than large-scale clinical evaluations. Along with the heterogeneity of study designs and technology configurations, these aspects must be considered when interpreting the overall evidence. Alongside these methodological aspects, the review highlights several challenges that remain relevant to the widespread adoption of 5G-based telemedicine, including the lack of protocol standardization, cybersecurity requirements, interoperability issues, and the need to strengthen the skills of healthcare personnel. Furthermore, in ultra-remote or latency-sensitive environments, it may be necessary to use hybrid configurations combining 5G and fiber-optic networks to ensure optimal performance. Overall, this analysis demonstrates that 5G has played a fundamental role in the evolution of telemedicine over the past decade, accelerating its transition towards a more accessible, efficient, and sustainable healthcare model. With the expansion of 5G coverage and integration with next-generation infrastructure, including fiber optics and future 6G networks, telemedicine is poised to become a fundamental component of healthcare systems, enabling high-quality specialist care even in underserved regions and supporting new models of distributed, real-time, and patient-centered healthcare.

## Figures and Tables

**Table 1:** Parameters extracted for telemedicine services from the included studies.

Application	Reference	Aim of the Study	N	Age	Dist. (km)	BW (Mbps)	Lat. (ms)	Service
Teleconsultation								
	Lubasch J.S. et al., 2024, [36]	User perception of practicality and institutional adoption of telemedicine devices.	350	n.s.	n.s.	n.s.	n.s.	Nursing home teleassistance
	Yang J. et al., 2024, [37]	Technical feasibility of 5G robotic teleultrasound for PCNL in complex renal stones.	15	42	5800	n.s.	177	Percutaneous neprolithotomy
	Wang C. et al., 2022, [38]	5G ultrasound remote consultation between provincial and primary care hospitals.	148	62.4 ± 15.7	n.s.	n.s.	n.s.	Medical education
	Tong L. et al., 2022, [39]	Cloud-based follow-up for patients with cardiac implantable electronic devices.	325	73.6 ± 10.7	n.s.	n.s.	n.s.	Surgical consultation
	Lu L. et al., 2022, [40]	Mixed reality (MR) technologies applied to orthopedic teleconsultation.	2	59–76	1300	n.s.	n.s.	Surgical consultation
	Zhang S. et al., 2022, [41]	Mobile internet-based mixed reality interactive telecollaboration (MIMIT).	4	n.s.	2489	n.s.	26	Surgical consultation
	Din N. et al., 2021, [42]	Intercontinental telementoring in corneal surgery.	3	n.s.	9263	10	1000	Surgical consultation
	Xie W. et al., 2021, [43]	AI-supported telementoring framework for congenital heart disease.	1	41	402	25	30	Surgical consultation
Telesurgery								
	Tian Y. et al., 2024, [44]	5G-assisted remote robotic lobectomy over ultra-long distance.	1	53	5000	n.s.	100	Lung cancer surgery
	Patel V. et al., 2024, [26]	Long-range low-latency connectivity evaluation in telesurgery.	5	n.s.	2600	n.s.	296 ± 50	Radical/partial nephrectomy
	Moschovas M.C. et al., 2024, [45]	Robotic telesurgery for prostate cancer treatment.	1	71	1300	n.s.	22	Robotic prostatectomy
	Alessio G. et al., 2024, [46]	5G-assisted telesurgery for epithelial basement membrane disease.	1	58	n.s.	n.s.	<50	PRK–PTK
	Li C. et al., 2024, [47]	Latency management system for 5G telesurgery.	15	n.s.	250	n.s.	n.s.	General surgery
	Li J. et al., 2023, [48]	Laparoscopic radical nephrectomy assisted by a remote robot.	29	63	187	n.s.	176	General surgery
	Moustris G. et al., 2023, [49]	Impact of 5G on telesurgical task performance (motion/video latency).	1	n.s.	3000	2	18/350	Surgical simulation
	Yang X. et al., 2022, [50]	Safety and reliability of 5G laparoscopic telesurgery.	1	71	3000	n.s.	254	General surgery
	Madder R.D. et al., 2021, [51]	Telestenting over regional vs. transcontinental distances.	1	n.s.	331–4965	n.s.	86–163	Simulation
	Zheng J.D. et al., 2020, [52]	Remote laparoscopic surgery using the “MicroHand” system.	4	n.s.	3000	1000	264	Simulation
	Tian W. et al., 2020, [53]	5G telerobotic spinal surgery feasibility (case series).	12	52.5	n.s.	n.s.	28	Spinal surgery
Telediagnosis								
	Jalayeri Nia G. et al., 2024, [54]	Home-delivered remote colon capsule endoscopy feasibility and satisfaction.	25	n.s.	n.s.	n.s.	n.s.	Endoscopy
	Han Z.-L. et al., 2024, [55]	Satisfaction determinants for 5G robotic ultrasound (SEM).	201	34–43	20	n.s.	n.s.	Ultrasound
	Liang W.H. et al., 2023, [56]	Practicality and safety of 5G robot-supported remote abdominal ultrasound.	39	7.5 ± 10.4	106	n.s.	180	Ultrasound
	Zhang Y.Q. et al., 2023, [57]	5G-supported telerobotic abdominal ultrasound on a remote island.	401	55.0 ± 15.4	72	930/130	200	Ultrasound
	He T. et al., 2023, [58]	Availability of 5G telerobotic ultrasound for breast assessments.	83	n.s.	72–220	930/130	200	Ultrasound
	Zhang T. et al., 2023, [59]	5G-based remote capsule endoscopy for stomach and small intestine.	20	46.4 ± 15.5	n.s.	n.s.	19.9	Endoscopy
	Ren J.-Y. et al., 2023, [60]	Large-scale evaluation of 5G-enabled robotic tele-ultrasound.	546	35.1 ± 10.3	29–40	240/45	<250	Ultrasound
	Saeki M. et al., 2022, [61]	Mobile ultrasound system for telemedicine in mountainous low-density areas.	5	n.s.	n.s.	15	1000	Ultrasound
	Duan S. et al., 2021, [62]	ICU robot-assisted tele-ultrasound feasibility and safety.	32	61 ± 20	n.s.	580/92	<200	Ultrasound
	Jing Wang et al., 2021, [63]	Robotic tele-echography for COVID-19 diagnosis over 5G.	1	87	700	930/132	n.s.	Ultrasound
	Ye R. et al., 2021, [64]	Robotic tele-echography for COVID-19 cardiopulmonary assessment.	23	n.s.	n.s.	930/132	n.s.	Ultrasound
	Duan B. et al., 2021, [65]	Description of a remote robotic ultrasound system with audio/video streaming.	95	n.s.	n.s.	n.s.	8	Ultrasound
	Wu S. et al., 2020, [66]	Bedside 5G robotic tele-ultrasound and remote consultation in COVID-19 regions.	4	62.8 ± 13.2	1756	930	20–30	Ultrasound
	Yu R. et al., 2020, [67]	Applications of 5G-supported remote robotic ultrasound for COVID-19.	2	45.5 ± 12.0	700	n.s.	n.s.	Ultrasound
Telemonitoring								
	Srichan C. et al., 2024, [68]	5G telehealth kiosk with non-invasive biosensors and forecasting for NCD indicators.	1844	n.s.	1.5–4	n.s.	n.s.	Glucose/BP monitoring
	Cheng W. et al., 2024, [69]	5G Smart Home Ward for chronic diseases monitoring and home care.	n.s.	n.s.	n.s.	n.s.	n.s.	Home monitoring
	Humayun M. et al., 2022, [70]	Multi-agent 5G framework (ABHCMF) for elderly/chronic patient monitoring.	4	n.s.	n.s.	n.s.	n.s.	Patient monitoring
	Angelucci A. et al., 2021, [71]	Continuous telemonitoring for chronic lung diseases leveraging 5G (Milan trial).	18	43.7	n.s.	n.s.	n.s.	Pulmonary monitoring
Teleassistance								
	Mammas C.S. et al., 2024, [72]	5G networks for trauma tele-assistance: ambulance monitoring and AI decision support.	6	n.s.	n.s.	10000	10	Emergency care
	Han W. et al., 2024, [73]	5G low-altitude air-to-ground collaborative medical rescue operations.	7	n.s.	60–600	n.s.	≤30	Medical emergency
	García F.M. et al., 2023, [74]	Health-5G mixed reality emergency assistance case study.	1	n.s.	n.s.	n.s.	300	Emergency response
	Xiang T. et al., 2023, [75]	5G-enabled prehospital emergency management solution.	1	68	n.s.	n.s.	n.s.	Chest pain triage
Telerehabilitation								
	Li X. et al., 2023, [76]	Center-based vs. cloud-based cardiac rehabilitation outcomes and satisfaction.	50	66.3 ± 4.0	n.s.	n.s.	n.s.	Cardiac rehabilitation
	Saeki M. et al., 2022, [61]	Remote rehabilitation/telehealth using mobile ultrasound in sparsely populated areas.	5	n.s.	n.s.	15	1000	Rehabilitation
	Oyama S. et al., 2022, [77]	Markerless motion capture integrated into low-latency IMT-2020 (5G) network.	5	n.s.	n.s.	n.s.	n.s.	Rehabilitation
	Han Y. et al., 2021, [78]	Remote follow-ups and programming for spinal cord stimulation (SCS) in chronic pain.	72	58.6 ± 1.6	800	n.s.	n.s.	Telestimulation

Notes: N = number of subjects; Age = mean subject age (years); Dist. = doctor–patient distance (km); BW = bandwidth (Mbps); Lat. = network latency (ms); n.s. = not specified.

## Data Availability

No new data were created.

## Supplementary Materials

The following supporting information can be downloaded at: https://www.mdpi.com/article/10.3390/bioengineering13040438/s1, PRISMA 2020 Checklist for systematic reviews.
